# Over-expression of miR172 causes loss of spikelet determinacy and floral organ abnormalities in rice (*Oryza sativa*)

**DOI:** 10.1186/1471-2229-9-149

**Published:** 2009-12-17

**Authors:** Qian-Hao Zhu, Narayana M Upadhyaya, Frank Gubler, Chris A Helliwell

**Affiliations:** 1 CSIRO Plant Industry, GPO Box 1600, Canberra, ACT 2601, Australia

## Abstract

**Background:**

Regulation of gene expression by microRNAs (miRNAs) plays a crucial role in many developmental and physiological processes in plants. miRNAs act to repress expression of their target genes via mRNA cleavage or translational repression. Dozens of miRNA families have been identified in rice, 21 of which are conserved between rice and Arabidopsis. miR172 is a conserved miRNA family which has been shown to regulate expression of *APETALA2 *(*AP2*)-like transcription factors in Arabidopsis and maize. The rice genome encodes five *AP2*-like genes predicted to be targets of miR172. To determine whether these rice *AP2*-like genes are regulated by miR172 and investigate the function of the target genes, we studied the effect of over-expressing two members of the miR172 family on rice plant development.

**Results:**

Analysis of miR172 expression showed that it is most highly expressed in late vegetative stages and developing panicles. Analyses of expression of three miR172 targets showed that *SUPERNUMERARY BRACT *(*SNB*) and *Os03g60430 *have high expression in developing panicles. Expression of miR172 was not inversely correlated with expression of its targets although miR172-mediated cleavage of *SNB *was detected by 5' rapid amplification of cDNA ends (RACE). Over-expression of miR172b in rice delayed the transition from spikelet meristem to floral meristem, and resulted in floral and seed developmental defects, including changes to the number and identity of floral organs, lower fertility and reduced seed weight. Plants over-expressing miR172b not only phenocopied the T-DNA insertion mutant of *SNB *but showed additional defects in floret development not seen in the *snb *mutant. However *SNB *expression was not reduced in the miR172b over-expression plants.

**Conclusions:**

The phenotypes resulting from over-expression of miR172b suggests it represses *SNB *and at least one of the other miR172 targets, most likely *Os03g60430*, indicating roles for other *AP2*-like genes in rice floret development. miR172 and the *AP2*-like genes had overlapping expression patterns in rice and their expression did not show an obvious negative correlation. There was not a uniform decrease in the expression of the *AP2*-like miR172 target mRNAs in the miR172b over-expression plants. These observations are consistent with miR172 functioning via translational repression or with expression of the *AP2*-like genes being regulated by a negative feedback loop.

## Background

microRNAs (miRNAs) are regulatory small RNAs that have important roles in regulating development and stress responses in plants [1-4]. They repress gene expression by targeting cognate messenger RNAs (mRNAs) for cleavage or translational repression [5,6]. Since the identification of the first rice miRNAs, based on sequence conservation with Arabidopsis [7], many new rice miRNAs have been identified using high-throughput small RNA sequencing approaches; the majority of these newly identified miRNAs are rice-specific [8-12]. miR172 is conserved in higher plants and has been shown to regulate expression of a sub-group of *APETALA2 *(*AP2*)-like transcription factors that contain two AP2 domains in Arabidopsis [13,14], tobacco [6] and maize [15-17].

In Arabidopsis, miR172 serves as a negative regulator of *AP2 *to specify floral organ identity. Over-expression of miR172 causes floral homeotic phenotypes similar to *ap2 *loss-of-function mutants [18], such as conversion of sepals and petals into carpels, and reduction of stamen numbers [14]. Expression of a miR172-resistant version of *AP2 *increases stamen number [19]. Arabidopsis miR172 also acts as a repressor of the *AP2*-like genes, *TARGET OF EAT 1 *(*TOE1*), *TOE2 *and *TOE3 *to promote early flowering [13,20]. miR172-mediated cleavage of mRNAs of these target genes has been detected [21], but there is strong evidence to suggest that the primary mode of repression of these target genes by miR172 is translational inhibition [13,14]. In turn, the transcription of miR172 target genes is under direct or indirect feedback regulation by their protein products [21].

In maize, expression of *GLOSSY15 *(*GL15*), an *AP2*-like gene with an mRNA targeted for cleavage by miR172, is gradually down-regulated during the early stages of vegetative development due to a progressive increase of miR172 levels, promoting the juvenile-to-adult transition [17]. Another two *AP2*-like paralogs, *INDETERMINATE SPIKELET1 *(*IDS1*) and *SISTER OF INDETERMINATE SPIKELET1 *(*SID1*), play multiple roles in inflorescence architecture in maize. Loss-of-function mutants of *IDS1 *lose spikelet determinacy and generate multiple florets [22]. No mutant phenotype has been observed in single *sid1 *mutants, but *ids1 sid1 *double mutants produce fewer tassel branches and generate multiple bracts in place of florets [16]. The *ids1 sid1 *double mutants rescue the phenotypic defects of *tasselseed4 *(*ts4*), a loss-of-function mutant of *MIR172e *[16], one of the five *MIR172 *genes in maize. This result suggests that both *IDS1 *and *SID1 *are targets of miR172. It has been shown that *IDS1 *and *SID1 *are regulated at the level of translation and transcript stability, respectively [15,16], indicating that a single miRNA can act in different ways on closely related mRNAs. The maize flowering-time gene *ZmRap2.7 *is closely related to Arabidopsis *TOE1*. Over-expression of *ZmRap2.7 *results in delayed flowering, while knock-down of this gene leads to early flowering [23]. However, it is not known whether or not *ZmRap2.7 *is also regulated by miR172 as *TOE1 *is in Arabidopsis.

The rice miR172 family contains four members (*MIR172a-d*), which are predicted to target five *AP2*-like genes, *Os03g60430*, *Os04g55560*, *Os05g03040*, *Os06g43220 *and *Os07g13170 *[ref [24] and this study]. *Os07g13170 *(*SNB *- *SUPERNUMERARY BRACT*) has been shown to be required for the correct timing of the transition from spikelet to floral meristem and for determination of floral organ identity. The T-DNA insertion mutant of *SNB *generates additional bracts (equivalent to rudimentary glumes) before development of a floret and also shows defects in floral organ development [24]. *SNB*, *Os03g60430, Os05g03040 *and *Os06g43220 *are the putative rice orthologs of maize *SID1*, *IDS1*, *ZmRap2.7 *and *GL15*, respectively [16].

We characterized the expression of miR172 and its putative *AP2*-like target genes in rice and did not find inversely correlated expression patterns although at least three of the *AP2*-like mRNAs were found to be cleavage targets of miR172, suggesting roles of miR172 via transcriptional and translation repression with the latter as a possible predominant mode of action of miR172 in rice. To investigate the functions of the *AP2*-like genes, we studied the effect of elevated expression of miR172 on rice development. Over-expression of miR172b recapitulates the phenotypes of *snb *and also gives rise to additional developmental defects not seen in *snb*. These results suggest that *SNB *and at least one of the other *AP2*-like target genes are down-regulated in plants over-expressing miR172b, indicating that other members of the *AP2*-like gene family also have roles in rice floret development.

## RESULTS

### Expression profiles of miR172 and its target genes

**Figure 1:**
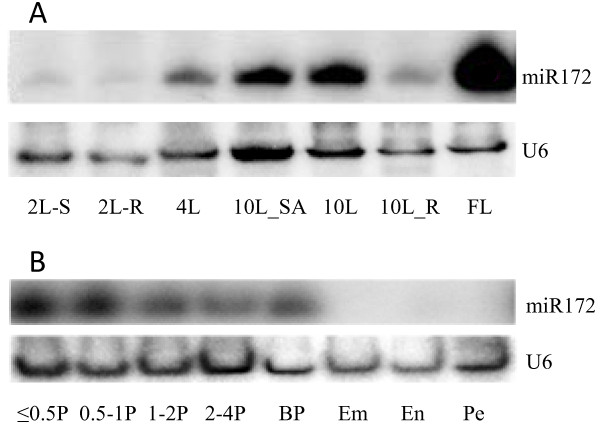
**RNA gel blot analysis of accumulation of miR172 in wild-type plants**. A, Accumulation of miR172 in vegetative tissues. 2L-S and 2L-R: shoot and root of two-leaf stage seedlings. 4L: the 4^th ^leaf. 10L: the 10^th ^leaf. 10L-SA: shoot apex of 10-leaf stage seedlings. 10L-R: 10-leaf stage root. FL: flag leaf. B, Accumulation of miR172 in reproductive tissues and grains. ≤ 0.5P, 0.5-1P, 1-2P and 2-4P: developing panicles with a length of ≤ 0.5 cm, 0.5-1 cm, 1-2 cm and 2-4 cm, respectively. BP: booting panicle. Em, En and Pe: embryo, endosperm and pericarp of 10 DAF grains, respectively.

To determine where miR172 and its target transcripts are expressed during rice development, we analyzed miR172 expression by RNA gel blot and expression of the *AP2*-like target mRNAs by qRT-PCR in various tissues. The mature miR172a-d sequences differ only in their 5' and 3' bases and therefore hybridization with a miR172a probe is likely to detect expression of all mature miR172 sequences. In wild-type plants, miR172 expression varied considerably between organs and developmental stages. Mature miR172 accumulation increased significantly in leaves but not in roots as plants grew, reaching a maximum in the flag leaf (Figure 1A). Similar expression patterns of miR172 have also been observed in vegetative tissues of Arabidopsis and maize [13,17], suggesting that miR172 has a conserved role during vegetative development. In reproductive tissues, miR172 was consistently expressed although its abundance reduced gradually during panicle development (Figure 1B). Expression of miR172 was below the detection limit in 10 DAF (days-after-fertilization) grains (Figure 1B). Higher expression of miR172 in later stage vegetative tissues and developing young panicles is consistent with a role in regulating the timing of floret initiation and development in rice.

**Figure 2:**
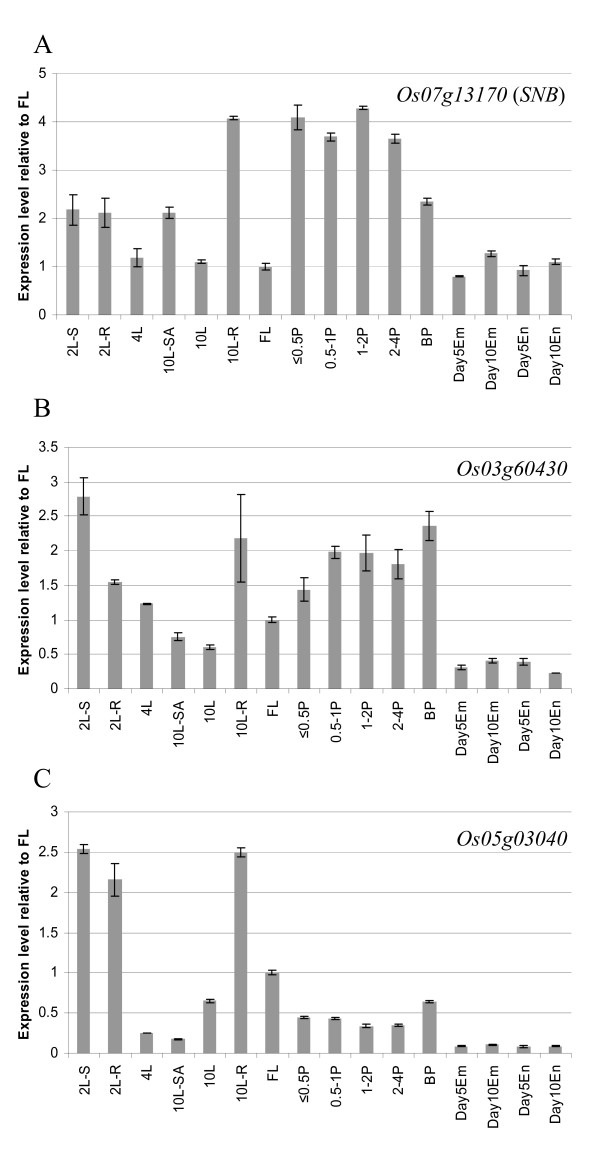
**qRT-PCR analyses of miR172 target genes in wild-type plants**. A primer pair spanning the miR172 target site was used to quantify expression of the uncleaved target mRNAs. For each gene, relative fold expression difference is shown by using the expression level detected in flag leaf as the reference. Error bars represent standard deviation of the expression ratio. 2L-S and 2L-R: shoot and root of two-leaf stage seedlings. 4L: the 4^th ^leaf. 10L: the 10^th ^leaf. 10L-SA: shoot apex of 10-leaf stage seedlings. 10L-R: 10-leaf stage root. FL: flag leaf. ≤ 0.5P, 0.5-1P, 1-2P and 2-4P: developing panicles with a length of ≤ 0.5 cm, 0.5-1 cm, 1-2 cm and 2-4 cm, respectively. BP: booting panicle. Em: embryo. En: endosperm.

The abundance of intact transcripts of miR172 target genes was analyzed by qRT-PCR using primer pairs spanning the miR172 cleavage sites. Expression of *SNB *(*Os07g13170*) was highest in developing panicles (<4 cm in length), in which differentiation of the spikelet and floral organs is progressing; expression of *SNB *was also high in roots from 10-leaf plants (Figure 2A). *Os03g60430 *was highly expressed in developing panicles and also in young seedlings (2L-S) (Figure 2B). In contrast expression of *Os05g03040 *was highest in young seedlings and roots (Figure 2C). All these three genes had a very low expression level in embryo, endosperm and pericarp of 10 DAF grains (Figure 2A, B, C). Expression of *SNB *and *Os03g60430 *showed an inverse correlation with the abundance of miR172 in two-leaf shoots, leaf four and leaf ten, but generally the expression of miR172 was not inversely correlated with the expression of its targets in the tissues analyzed (Figure 2A, B, C). We were unable to specifically amplify *Os06g43220 *even though several primer combinations were tried; this could be a result of very low expression (supported by the relatively low number of ESTs found in both japonica and indica rice; data not shown). We were unable to quantify *Os04g55560 *expression as the gene-specific product was always accompanied by a non-specific product. These expression profiles support previous results showing that *SNB *has a role in controlling spikelet determinacy and floret development [24], and also suggest that *Os03g60430 *could play a role in floret development.

### miR172-mediated cleavage of target genes

**Figure 3:**
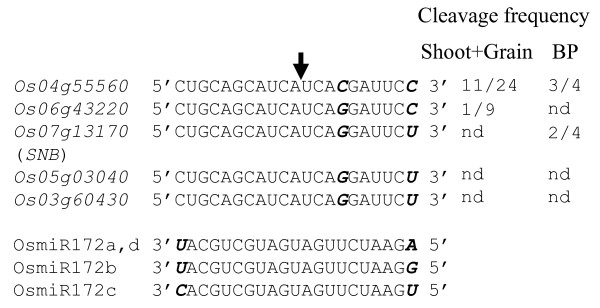
**Analysis of miR172-mediated cleavage of target genes**. 5' RACE was used to map the miR172-mediated cleavage sites in the predicted targets. The expected cleavage site is indicated by an arrow. Nucleotides that differ among miR172 family members or their targets are shown in bold italic. The cleavage frequencies (number of clones with the expected cleavage site/total number of clones sequenced) detected in the indicated tissues are shown to the right of the sequence alignment. BP: booting panicle. nd: no RACE product detected.

miR172 has been shown to cleave *AP2 *and *AP2*-like target mRNAs in Arabidopsis [13,14,21] and maize [15,17], but is thought to act predominantly through translational repression [13-15]. To determine whether the five putative targets of miR172 in rice are cleaved by miR172, 5' rapid amplification of cDNA ends (RACE) analysis was performed using RNA isolated from two-leaf stage shoots, 1-10 DAF grains and booting panicles (BP). Cleavage of *Os04g55560 *was detected in a mixed sample of shoot and grain as well as in booting panicles; cleavage of *Os06g43220 *was only detected in the mixed sample with a low frequency (most likely contributed by young seedlings as accumulation of miR172 was below the detection limit in 10 DAF grains); and cleavage of *SNB *was only detected in booting panicles. No cleavage was detected for *Os03g60430 *or *Os05g03040 *in any of the samples analyzed (Figure 3). These results suggested tissue- or cell-type-specific expression of miR172 and/or its target genes.

### Over-expression of miR172b delays the transition from spikelet meristem to floral meristem

**Figure 4:**
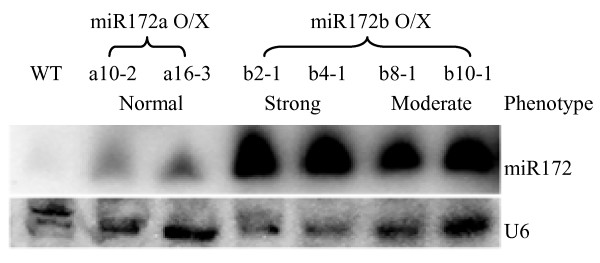
**RNA gel blot detection of accumulation of miR172 in mature leaves of wild-type and miR172 transgenic plants**. a10-2 and a16-3 were transformed with pre-*MIR172a *and had normal phenotype; b2-1 and b4-1 were transformed with pre-*MIR172b *and showed strongly altered phenotypes; b8-1 and b10-1 were also transformed with pre-*MIR172b *but showed moderately altered phenotypes. O/X: over-expressor.

**Figure 5:**
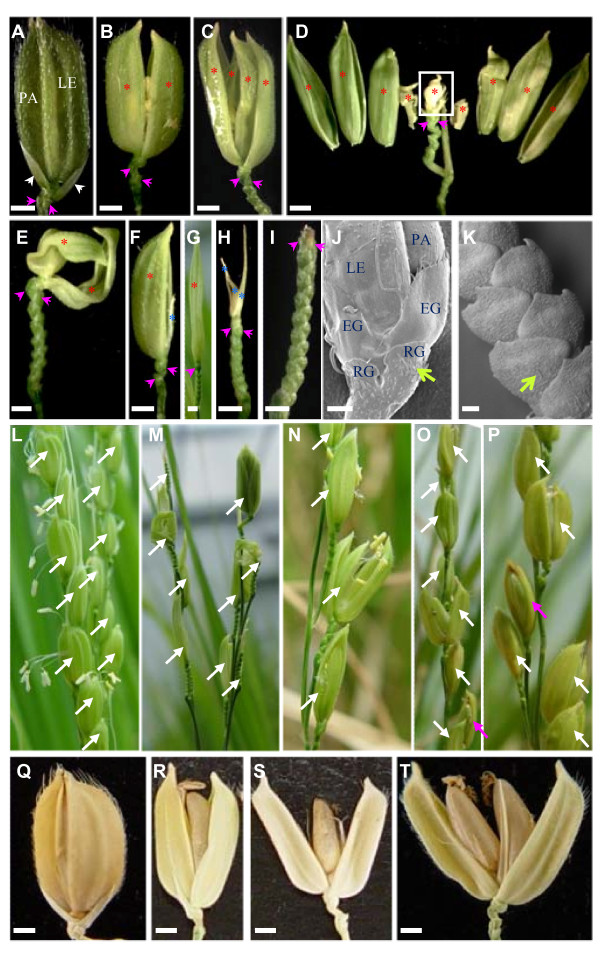
**Phenotypes of spikelets and mature seeds of plants over-expressing miR172b**. A, A wild-type spikelet comprising a single floret enclosed by lemma and palea, a pair of rudimentary glumes (indicated by pink arrow heads) and a pair of empty glumes (indicated by white arrow heads). B to I, Individual spikelets from plants over-expressing miR172b. All of these spikelets do not have empty glumes but have multiple bract-like structures (top pair indicated by a pair of pink arrow heads). B, Fertile spikelet with a pair of lemma and palea (indicated by red *, same for C to G). C, Sterile spikelet with two pairs of lemma and palea. D, Sterile spikelet with multiple pairs of lemma and palea. The boxed part still contains multiple layers of lemma and palea. E, Sterile spikelet with a pair of twisted lemma and palea. F, Sterile spikelet with a normal-looking lemma or palea and a degenerated lemma or palea (indicated by blue *, same for H). G, Sterile spikelet with elongated lemma or palea. H, Sterile spikelet with multiple layers of degenerated lemma or palea. I, Sterile spikelet without floret. J, Scanning electron micrograph (SEM) of wild-type spikelet to show the surface features of empty glumes and rudimentary glumes. K, SEM of the bract-like structures of a spikelet from a miR172b over-expression plant. Green arrows in J and K indicate trichomes. L, Part of a wild-type panicle with normal spikelets. M, Part of a panicle from a miR172b over-expression plant with a strongly altered spikelet phenotype. N to P, Part of panicles from a miR172b over-expression plant with a moderately altered spikelet phenotype. White arrows in L to P indicate individual spikelets. Spikelets indicated by pink arrows in O and P represent spikelets with degenerated lemma or palea. Q, Wild-type mature seed. R to T, Mature seeds from plants over-expressing miR172b showing naked single grain (R and S) or double grains (T). EG: empty glume. LE: lemma. PA: palea. RG: rudimentary glume. Bars in A to I, and Q to T are 1 mm. Bars in J and K are 100 μm.

We generated transgenic plants expressing the stem-loop precursors of miR172a and miR172b, transcribed by the maize ubiquitin promoter. Elevated levels of miR172 were detected in these miR172 over-expression plants, particularly in plants transformed with pre-*MIR172b *(Figure 4). Transgenic plants over-expressing miR172b showed normal vegetative growth and heading time, but the inflorescence (panicle) of the transformed plants was smaller, producing the half number of primary branches of the untransformed wild-type (Table 1). A wild-type spikelet consists of a single floret and two subtending pairs of bract-like structures - a pair of rudimentary glumes and a pair of empty glumes (Figure 5A, J). Spikelets of plants over-expressing miR172b were generated from primary or secondary branches as in wild-type, but the majority of spikelets were abnormal and showed variable defects in floral organs. The common phenotypes of the mutated spikelets were that more than two, and in extreme cases as many as 20, bract-like structures were generated before transition to floral development (Figure 5B to 5H); in some cases no obvious floral organs were produced (Figure 5I). The majority of these spikelets lacked a pair of empty glumes (compare Figure 5A with Figure 5B to 5H; Table 2). Scanning electron microscopy (SEM) showed that the lower part of the rudimentary glumes in the wild-type plant had round projections and small trichomes (Figure 5J). In the miR172b over-expression plants, similar round projections coated the surface of the bract-like structures though fewer trichomes were seen (Figure 5K), suggesting that the additional bract-like structures in the miR172b over-expression lines have the same identity as rudimentary glumes. This result suggests that reproductive development of plants over-expressing miR172b was not affected until the formation of the spikelet meristem, but the transition from spikelet meristem to floral meristem was delayed, leading to the reiteration of bract-like structures.

**Table 1:** Phenotype scores of plants over-expressing miR172b

Trait	Moderate phenotype plants	Strong phenotype plants	Wild-type
Number of primary branches	4.4 ± 0.2	3.7 ± 0.2	8.9 ± 0.9
Abnormal seed (%)	95.7 ± 2.1	100 ± 0	0
Severely degenerated spikelet (%)	5.8 ± 3.5	21.8 ± 14.6	0
Fertility (%)	40.1 ± 5.0	1.9 ± 2.9^a^	97.5 ± 2.9
Weight of structurally normal seed (g)	2.27 ± 0.03	na^b^	2.46 ± 0.09
Weight of abnormal seed (g)	1.84 ± 0.16	1.80 ± 0.12	na

^a^The range of fertility was between 0 and 7.6%.

^b^na: not applicable.

**Table 2:** Number of floral organs in plants over-expressing miR172b

No. of organs	Bract^a^		Empty glume		Lemma+Palea		Lodicule		Stamen		Carpel	
	
	S^b^	M^b^	S	M	S	M	S	M	S	M	S	M
0			100	173								
1			19	47				1			17	198
2		8	1	16	2	83	7	145			3	6
3		51			18	85	13	23	5	8		
4	15	77				36		25	15	14		
5	8	31						5		37		
6		22						3		92		
7		16								7		
8		21						2		1		
9	2	5										
>=10	95	5										

Number of spikelet checked	120	236	120	236	20^c^	204^c^	20^c^	204^c^	20^c^	204^c^	20^c^	204^c^

Number of floral organ in WT	2		2		2		2		6		1	

^a^Equivalent to rudimentary glume in the wild-type (WT).

^b^S (strong) and M (moderate) phenotype plants.

^c^Excluding spikelets with four or more layers of lemma- and palea-like structures but no other distinguishable floral organs.

### Over-expression of miR172b reduces fertility and seed weight

Plants over-expressing miR172b showed significant floret defects and reduced fertility (0-44.1%) compared to wild-type. Based on the number of deformed spikelets and degree of fertility, plants over-expressing miR172b could be grouped into strong (Figure 5M) and moderate (Figure 5N, O, P) phenotypes. Plants with <10% fertility and >10% severely degenerated spikelets were defined as having a strong phenotype, with the remainder classified as moderate phenotype plants. Spikelets without obvious floral organs (Figure 5I), or with several layers of small lemma- and palea-like structures but without distinguishable internal reproductive organs (Figure 5H) were classed as severely degenerated spikelets. The percentage of severely degenerated spikelets was as high as 45% in some strong phenotype plants. In addition, the remaining spikelets of strong phenotype plants were also significantly deformed (Figure 5M), with phenotypes including multiple layers of lemma and palea (Figure 5C, D), twisted lemma and palea (Figure 5E), degeneration of either lemma or palea (Figure 5F), or leaf-like structures replacing lemma and palea (Figure 5G). All of these deformed spikelets were sterile, and as a consequence, most strong phenotype plants were completely sterile. Some strong phenotype plants set a small number of fertile spikelets but none of them had a wild-type appearance (Table 1). On average, the moderate phenotype plants had ~6% severely degenerated spikelets and ~40% fertility (Table 1), but less than 5% of the fertile spikelets were essentially normal, i.e. with a pair of empty glumes and normal lemma and palea. Analysis of miR172 expression showed that plants with the strongest phenotypic aberrations had the highest expression levels of miR172 (Figure 4).

The common features of fertile but abnormal spikelets were that they had four or fewer lemma- and palea-like structures, and that part of or even the whole of the grain was naked due to failure of the lemma and palea to close after flowering (Figure 5R, S, T) or because of degeneration of these structures (Figure 5O, P). The weight of these seeds was reduced compared to wild-type seeds (Table 1), with the most naked seeds showing the greatest reduction (Figure 5S). This suggests that closing of lemma and palea may be important for optimal grain filling and maturation in rice.

### Over-expression of miR172b results in homeotic transformation and other changes of floral organs

**Figure 6:**
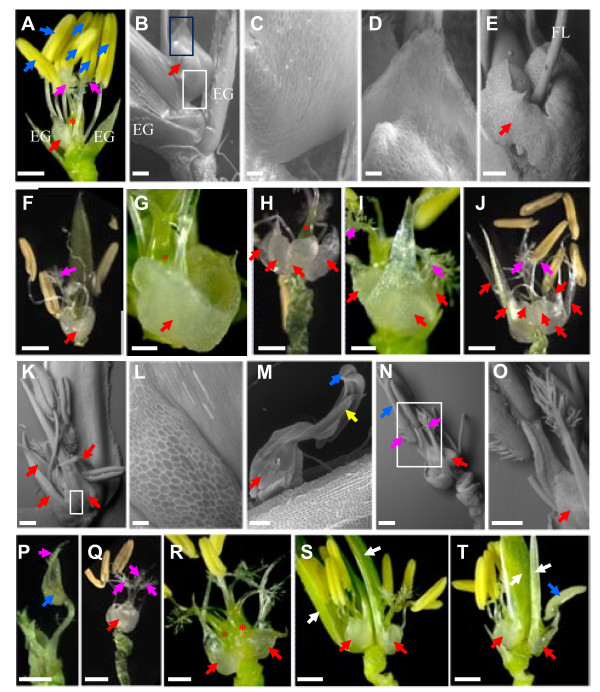
**Changes in the number of floral organs and floral identity in plants over-expressing miR172b**. A, A wild-type floret with lemma and palea removed to show lodicule (only one is seen which is indicated by a red arrow), six anthers (indicated by blue arrows), one carpel (indicated by a red *) and two stigmas (indicated by light pink arrows). B, SEM of the basal part of a wild-type spikelet showing the morphology and surface features of a lodicule. C and D, Close-ups of the lodicule in the white and blue boxed regions in B, respectively. E to T, Images of floret or floral organs of plants over-expressing miR172b. E, Two swollen lodicules fused together. F and G, Two enlarged lodicules fused together to form a cup-like structure. H, Multiple lodicules located at the same whorl. I and J, Multiple lodicules located at two whorls with elongated lodicules at the outer whorl. K, All four lodicules are elongated and show distinct features in the middle and flanking regions at the base. L, A close-up of the white boxed region shown in K. M, An elongated lodicule (tip of the lodicule is indicated by a yellow arrow) fused with an anther (indicated by a blue arrow). N, Lodicule base and anther top organ replaced carpel and completely separated two stigmas that showed flat style. O, A close-up of the boxed portion in N. P, Conversion part of the stigma into an anther. Q, Floret with three stigmas. R, Floret with two carpels (indicated by red *). S and T, Two florets developed within a single spikelet and one of them (left side one) always with incomplete floral organs. White arrows indicate lemma or palea. To show the internal floral organs, both lemma and palea (for A, B, E, G to J and N to R) or one of them (for F, K, S and T) has been removed. EG: empty glume, FL: filament. Bars in A, F, H to J, and P to T are 1 mm. Bars in B, E and G are 200 μm. Bars in C, D and L are 42 μm. Bars in K, M to O are 420 μm.

The unit comprising lemma, palea and floral organs including two lodicules on the lemma side, six anthers and a carpel with two stigmas is called floret (Figure 6A). Wild-type lodicules have a wide base, a rough surface and a narrow apex (Figure 6B, C, D). When flowering, the floret opens due to swelling of the lodicules, and closes after a few minutes (depending on temperature and humidity) due to shrinking of the lodicules. In plants over-expressing miR172b, florets with five or more layers of lemma and palea could not open due to the tightly closed lemma and palea. Florets that did not completely close up after flowering had altered numbers and/or morphology of lodicules. One (due to fusion of two lodicules; Figure 6E, F, G) to as many as eight lodicules were observed (Table 2). Multiple lodicules were arranged in one (Figure 6H to 6K) or two (Figure 6I, J) whorls, with similar surface features to those of wild-type but swollen (Figure 6H), or elongated significantly and converted into a structure similar to the palea marginal region (Figure 6I, J, K). In the case of two whorls of lodicules, usually only the lodicule in the outer whorl was converted (Figure 6J). In the converted lodicules, two edges of the base section retained their original identity (Figure 6L), resulting in a mosaic floral organ. The most frequently observed mosaic floral organ was a lodicule base with an anther fused to the elongated lodicule apex (Figure 6M). Occasionally, a mosaic organ with a lodicule base and an anther top was observed at the innermost whorl of the floret, in which the mosaic organ replaced the carpel and the identities of two stigmas were also converted (Figure 6N, O). In some florets, a stigma was partially converted into an anther (Figure 6P). These results suggest that timing and/or positioning of the floral organ meristems are interrupted by over-expression of miR172b, indicating that a proper expression of miR172 target genes is important in specification of floral organ identities.

Stamens were also frequently altered in plants over-expressing miR172b. All florets of the strong phenotype plants and approximately half the florets of the moderate phenotype plants had less than the six stamens found in wild-type (Table 2). Usually, anthers of plants over-expressing miR172b were slightly smaller than those of wild-type, although no other obvious defects were observed. The carpel was the most stable floral organ, with <5% of spikelets developing two carpels (Figure 6R; Table 2). In some spikelets both carpels were fertilized and developed into normal-looking grains (Figure 5T). Occasionally, three stigmas were observed instead of two (Figure 6Q). Ectopic florets were found in ~10% of spikelets, a few of these developed incomplete internal floral organs (Figure 6S, T), none were fertile.

Plants transformed with pre-*MIR172a *did not show any altered phenotypes (data not shown), even though miR172 accumulated to a higher level than in wild-type plants (Figure 4).

### SNB mRNA abundance is not reduced in miR172b over-expression plants

**Figure 7:**
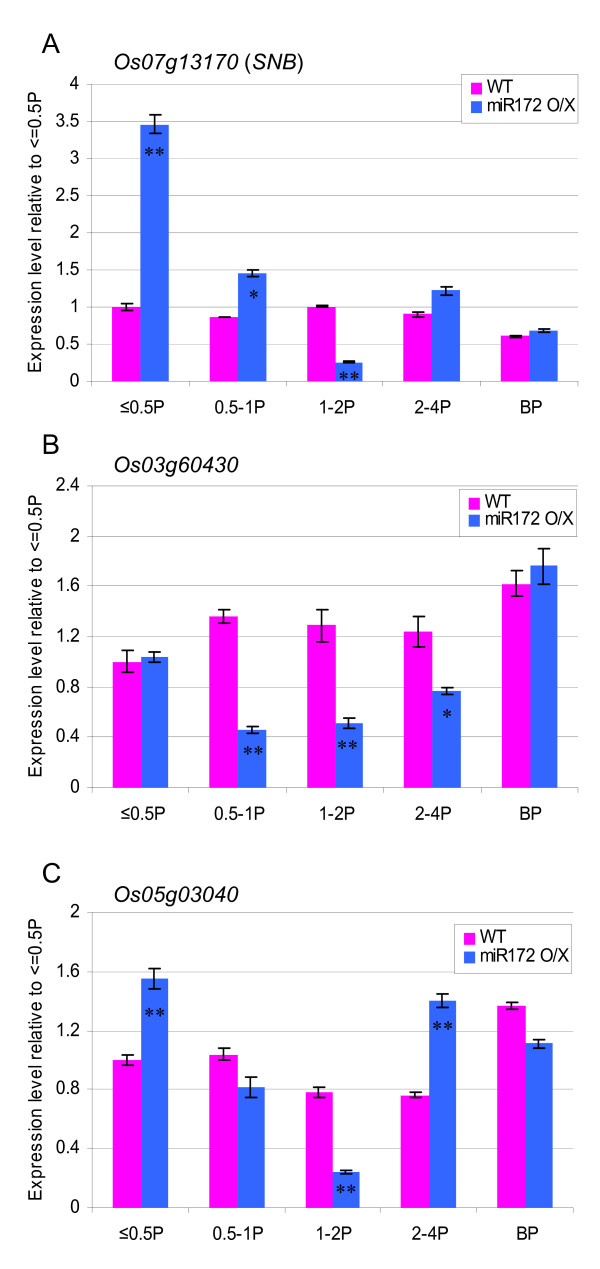
**qRT-PCR analyses of miR172 target genes in panicles of wild-type and miR172b over-expression plants**. Expression levels of each gene in various tissues were analyzed using a primer pair spanning the miR172 target site. For each gene, relative fold expression is shown by using the expression level detected in ≤ 0.5 cm long panicles of wild-type as the reference. The tissues where a significant increase or decrease of expression was detected in plants over-expressing miR172b compared to wild-type are indicated (* for *p *≤ 0.05 and ** for *p *≤ 0.01, based on student *t*-test). Error bars represent standard deviation of the expression ratio. WT: wild-type. O/X: over-expressor. ≤ 0.5P, 0.5-1P, 1-2P and 2-4P: developing panicles with a length of ≤ 0.5 cm, 0.5-1 cm, 1-2 cm and 2-4 cm, respectively. BP: booting panicle.

The phenotype observed in miR172b over-expression plants is consistent with reduced *SNB *function during panicle development. As *SNB *is cleaved by miR172 a reduced accumulation of *SNB *mRNA would be expected in miR172b over-expression plants. However, we observed more *SNB *mRNA accumulating in early stage panicles in these plants (Figure 7A). A similar effect was observed for *Os05g03040 *mRNA (Figure 7C). Reduced accumulation of the *Os03g60430 *mRNA was observed in panicles between 0.5 cm and 4 cm (Figure 7B), suggesting that the over-expression of miR172b can lead to increased cleavage of this transcript.

## Discussion

In this study, we have shown that over-expression of miR172b in rice resulted in i) a smaller panicle due to reduction of primary branches, ii) spikelets with multiple bracts resembling rudimentary glumes, iii) florets with multiple layers of lemma- and palea-like structures but without empty glumes, iv) abortion of inner floral organs, especially in spikelets with more than 10 bracts or four layers of lemma- and palea-like structures, v) changes in numbers, size, appearance, and identities of floral organs, especially lodicules and stamens, vi) ectopic florets, and vii) sterility and reduced seed weight. These phenotypes not only recapitulated but enhanced the mutant phenotypes of *SNB*, suggesting that *SNB *and at least one of the other four targets of miR172 were repressed in plants over-expressing miR172b. We provide direct evidence for miR172-mediated cleavage for *SNB*, *Os04g55560 *and *Os06g43220*. However, expression of *SNB *was not inversely correlated with expression of miR172 in wild-type, and over-expressing miR172b did not reduce the expression levels of *SNB *in <1 cm long panicles where development of spikelets and florets is occurring, instead *SNB *transcript abundance increased significantly. The unchanged or increased abundances of miR172 target mRNAs in the miR172b over-expression plants is reminiscent of observations made in Arabidopsis [13,21] where there is evidence that miR172 acts to repress translation and for transcription of the *AP2*-like genes to be under negative feedback regulation via their protein products. Our data cannot distinguish between these possibilities but do suggest a conservation of regulation of the *AP2*-like genes between Arabidopsis and rice.

### Control of spikelet determinacy and floret development in rice

Rice spikelets, initiated from primary or secondary branches of the inflorescence, have a determinate fate and consist of two rudimentary glumes and a single functional floret. Previously, *BRANCHED FLORETLESS1 *(*BFL1*) or *FRIZZY PANICLE *(*FZP*) and its maize ortholog *BRANCHED SILKLESS1 *(*BD1*) have been shown to be regulators of spikelet determinacy in rice and maize, respectively [25-27]. Knock-out mutants of *BFL1 *and *BD1 *fail to initiate floret meristems, and instead they continuously generate axillary branch meristems from the axils of rudimentary glumes to produce a highly branched inflorescence [25-27], indicating that they specify meristem identity during the transition from spikelet meristem to floral meristem. Recently, *SNB*, a target of miR172, has been shown to be another gene regulating this transition [24] with *snb *mutants producing multiple bract-like structures that are equivalent to rudimentary glumes. Our results show that *SNB *is a target of miR172, which adds another layer of complexity to the regulation of spikelet determinacy in rice. It has been proposed that *SNB *acts downstream or independentlyof *BFL1*, based on the phenotypes of the respective mutants and mRNA expression patterns determined by *in situ *hybridization [24,26]. However, further experiments are required to confirm this relationship.

*SNB *is required for the correct timing of the transition from spikelet meristem to floret meristem in rice as this transition is delayed in the *snb *mutants [24]. According to previous *in situ *results, *SNB *is initially expressed in the branch meristem and spikelet meristem, and is then primarily restricted in the boundary region of the spikelet and glume primordia. Once the spikelet meristem is converted into a floret meristem, a decreased expression of *SNB *was observed [24]. Our data showed that both miR172 and *SNB *are highly expressed in <1 cm long panicles, so miR172 could be acting to restrict the expression domain of *SNB*. However at present the precise expression domain of miR172 in the panicle is yet to be determined.

Phylogenetic analysis has shown that *SNB *and *Os03g60430 *are likely to be orthologous to maize *SID1 *and *IDS1*, respectively [15,16]. These genes together with the *Q *gene of wheat [28] appear to be grass specific and are involved in panicle and spikelet development. Single mutants of *SID1 *do not show visible phenotypic changes, but null mutants of *IDS1 *lose spikelet determinacy and produce extra lateral florets [22]. However, double mutants of *IDS1 *and *SID1 *continuously initiate multiple bracts and do not make any florets [16]. Thus, both *IDS1 *and *SID1 *are necessary for initiation of floral meristems. Both *snb *and the *ids1 sid1 *double mutants produce multiple bracts, but *snb *only occasionally produces bracts continuously [16,24], whereas plants with strongly over-expressed miR172b have an average of 22% of spikelets without floral organs (Table 1). We speculate that the additional floret defects observed in plants over-expressing miR172b are due to repression of *Os03g60430 *by over-expressed miR172 because both *SNB *and *Os03g60430 *are relatively highly expressed in developing panicles (Figure 2A, B), they have similar mRNA expression patterns determined by *in situ *hybridization [24,29], and *Os03g60430 *is down-regulated by elevated levels of miR172 in 0.5-4 cm long panicles (Figure 7B).

5' RACE results suggest that *Os04g55560 *is regulated by miR172 in both vegetative and reproductive tissues (Figure 3). Among the five miR172 targets in rice, *Os04g55560 *is most similar to Arabidopsis *AP2 *based on phylogenetic analysis, but its function has not been investigated in rice. In Arabidopsis, both loss-of-function *ap2 *mutants and miR172 over-expression plants have carpels in place of perianth organs (sepals and petals) due to the absence of *AP2 *and ectopic expression of *AGAMOUS *(*AG*), a class C gene, in the outer two whorls of the flower primordium [13,14]. We occasionally observed florets with two carpels or a carpel with multiple stigmas. In most florets multiple lodicules with changed morphology were seen. Lodicules are thought to be homologous to petals in eudicots. These phenotypic changes could be partly resulted from repression of *SNB *because the *snb *mutant also showed changes in lodicules [24]. Further investigation is required to determine whether these altered phenotypes are also related to changes in expression of *Os04g55560*.

### Functional specificity of miR172 members

Maize *MIR172e *loss-of-function mutants show increased inflorescence meristem branching and develop carpels within the tassel [15], indicating miR172e has a specific function. This could be a result of spatiotemporal expression differences between individual members of the miR172 family, or their targets, but does not rule out the possibility that only *MIR172e *is functional. Of the four rice *MIR172 *members, *MIR172b *has a mature miRNA sequence identical to maize *MIR172e*. In addition, the rice *MIR172b *and maize *MIR172e *are located in a syntenic region [15]; therefore, it is of interest to know whether *MIR172b *also plays a non-redundant role in inflorescence and spikelet development in rice and whether the other three members are expressed and functional in rice development.

Expression analysis of the mature miR172 sequences and their precursors in different tissues and developmental stages might help determine where and when each miR172 member is likely to be expressed; however, distinguishing expression of individual miR172 family members using hybridization and PCR-based approaches is difficult because the four miRNAs have few sequence differences. Small RNA sequencing is able to distinguish individual members with identical mature miRNAs due to differences in the miRNA* sequences. It has been shown that miR172b is expressed in seedlings and developing grains [8,10,12], whereas miR172c is not detected in developing grains [12]. miR172a/d is detected in seedlings and developing grains but the miRNA* is only detected for miR172d [[8,12]http://mpss.udel.edu/rice/]. These results suggest that miR172a might not be expressed in these two tissues. In our study, over-expression of *MIR172a *did not show any visible mutant phenotype. This might be because the accumulation of miR172 in the *MIR172a *over-expression plants was not sufficient to cause a phenotypic change (Figure 4). The reduced accumulation of miR172 could be because the transgene containing pre-*MIR172a *is transcribed less efficiently than the pre-*MIR172b *transgene, or as pre-*MIR172a *is the least stable precursor (ΔG = -49.1 kcal/mol) among the four miR172 precursors in rice, it may be cleaved by miR172a itself as shown in Arabidopsis [30]. In Arabidopsis, a miR172a miR172b (both with the same mature miRNA sequence as rice miR172a) double mutant does not show any floral defects (it is not clear whether the plants have other defects) [19]. Further work is needed to determine whether miR172a has a role in rice development.

## Conclusions

Over-expressing miR172b resulted in delayed transition from spikelet meristem to floret meristem and caused defects in floret development. This is a result of repression of *SNB *and at least one of the other four target genes, most likely *Os03g60430*, by the elevated levels of miR172 in plants over-expressing miR172b. Our analyses of expression of miR172 and its target mRNAs are consistent with it acting through transcriptional and/or translational repression with the latter as a possible predominant mode of action of miR172 in rice.

## Methods

### Plant materials and growing conditions

All experiments were performed using rice (*Oryza sativa spp*. japonica) cultivar Nipponbare. Rice tissue samples were collected from plants grown in a controlled glasshouse at 25 ± 3°C with 16 hours of light, except the two-leaf-stage shoots and roots that were collected from young seedlings grown in Petri dishes at 28°C. For miR172 over-expression transgenic lines, mature leaves (for northern blot) and panicle samples (for qRT-PCR) were collected from T_0 _plants. The two-leaf-stage shoot sample included shoot apices and all leaves. The 10-leaf-stage shoot apex sample included the basal ~0.5 cm part of young leaves that are ~1 cm in length. Two-, four- and ten-leaf-stage samples were used to represent juvenile, intermediate and adult vegetative stage, respectively. Panicles with a length of less than 0.5 cm and 0.5-4 cm represent differentiation stage of spikelets and florets, respectively. Booting panicle was representative of developed panicle.

### Generation of miR172 over-expression constructs and transgenic plants

The genomic sequences containing pre-*MIR172a *or pre-*MIR172b *were amplified using locus-specific primers. For the *MIR172a *locus, the primers were 5'-GAGCTCCATGGATGGAACGGTAGAGTCGGTGT-3' and 5'- GAGCTCGTATGGTCTTTGAATAGCAGAGGAGC-3'. For the *MIR172b *locus, the primers were 5'-GAGCTCCAGTAGAGAGTGTGATGCCGCAGCT-3' and 5'-GAGCTCGCGGCGTTGGTACAATTAAGCTGATG-3'. The first six nucleotides in each primer formed a *Sac*I restriction site. The PCR fragments were cloned into pGEM^®^-T Easy vector (Promega, Madison, WI). To generate the ubiquitin-pre-*MIR172 *constructs, the *Sac*I fragment released from the pGEM^®^-T Easy vector was gel purified and cloned into the similarly digested vector pKU352 [31]. Rice transformation was performed by the *Agrobacterium tumefaciens*-mediated co-cultivation approach as described previously [32]. Transformed calli were selected on hygromycin-containing media.

### RNA isolation, qRT-PCR analysis and miR172-mediated cleavage of target genes

Total RNA was isolated as described previously [12]. Ten micrograms of total RNA was treated with 10 units of RQ1 RNase-free DNase (Promega, Madison, WI), and purified by phenol-chloroform extraction. Five micrograms of DNase-treated total RNA was used in both reverse transcription (RT) reactions and no RT controls. First-strand cDNA was synthesized by random primer using the SuperScript III RT kit (Invitrogen, Carlsbad, CA) following the manufacturer's instruction.

qRT-PCR analyses were carried out using an ABI 7900 HT Fast Real-Time PCR System **(**Applied Biosystems, Foster City, CA). For each PCR, 5 μl of 1:40 diluted template cDNA was mixed with 1 μl of 10 × PCR buffer, 0.7 μl of 50 mM MgCl_2_, 0.4 μl of 5 mM dNTPs, 0.4 μl each of 10 mM forward and reverse primers, 0.5 μl of 1:10000 diluted SYBR and 0.1 μl of platinum *Taq *DNA polymerase (Invitrogen, Carlsbad, CA) and 1.5 μl of DEPC dH_2_O to a final volume of 10 μl. The amplification program was: 15" at 95°C, followed by 15" at 95°C, 15" at 60°C and 45" at 72°C for 35 cycles, and then followed by a thermal denaturing step to generate dissociation curves to verify amplification specificity. All reactions were performed using one biological sample with at least three technical replicates, and the sizes of the PCR products were validated by electrophoresis on a 1.5% agarose gel. Rice 18S rRNA was used as control for internal normalization because it was found to be uniformly expressed in the tissues used in this study. PCR efficiencies were calculated using the LinRegPCR program http://www.gene-quantification.de/download.html#linregpcr. Relative expression analyses were based on Pfaffl (2001) [33]. Primers used are listed in Table 3.

**Table 3:** Primers used in this study

Primer name	Sequence	Usage
Os03g60430_RTF	5'-GGGCTCGTCTCCCCAATGGACT-3'	qRT-PCR
Os03g60430_R1	5'-GGTGTTTCACCGGCAAGGCGAT-3'	5' RACE
Os03g60430_R2	5'-TCAGGCGGTTGGCGGGAAGTAGAA-3'	qRT-PCR and 5' RACE
Os04g55560_R1	5'-GCATCCAGCTCTTGTTCTTGCTGGTA-3'	5' RACE
Os04g55560_R2	5'-AGGTGGGCCGGGTCAGGGAATGG-3'	5' RACE
Os05g03040_RTF1	5'-GACTGCCCAACCTCATCCCCTAT-3'	qRT-PCR
Os05g03040_R1	5'-TGGGCGTTTTATGTGTGGATGCAA-3'	qRT-PCR and 5' RACE
Os05g03040_R2	5'-GGTGGTGGTGATGGCGGCTTGA-3'	5' RACE
Os06g43220_R1	5'-GGGGAACATCAGGTCGTCGGCTT-3'	5' RACE
Os06g43220_R2	5'-CTGCAGCTAAGAAGAATCCTA-3'	5' RACE
Os07g13170_RTP1	5'-ATGGAAGGGAAGCTGTTACT-3'	qRT-PCR
Os07g13170_R1	5'-CAGGTGGAACATAGAGAGGGATA-3'	5' RACE
Os07g13170_R2	5'-TCAGGCGGTCGGGGGGAAGTAGAA-3'	qRT-PCR and 5' RACE
18S_F	5'-ATGATAACTCGACGGATCGC-3'	qRT-PCR
18S_R	5'-CTTGGATGTGGTAGCCGTTT-3'	qRT-PCR

5' RACE was used to analyze cleavage of the predicted target genes of miR172 following the approach described previously [12].

### Northern blot hybridization analysis

Approximately 30 μg of total RNA was separated on 18% polyacrylamide denaturing gels, using a rice miR172a RNA oligonucleotide as a size marker. RNAs were transferred to Amersham Hybond™-N^+ ^membrane (GE Healthcare, Amersham, UK) and hybridized with a locked nucleic acid DNA oligonucleotide complementary to the miR172a sequence, which had been T4 kinase labelled with γ-^32^P ATP. Blots were prehybridized and hybridized at 42°C in 125 mM Na_2_HPO_4 _(pH 7.2), 250 mM NaCl_2_, 7% SDS and 50% formamide, and washed at 42°C twice with 2 × SSC, 0.2% SDS followed by a higher stringency wash of 1 × SSC, 0.1% SDS at 37°C if required. Blots were imaged using an FLA-5000 phosphorimager (Fuji Medical Systems Inc. USA). U6 was used as a loading control.

### Scanning electron microscopy observations

Spikelets from the wild-type and miR172b over-expression plants were fixed in 70% ethanol for two hours. After dehydration through an ethanol series, the samples were dried to a critical point and mounted on stubs, and then were examined with a scanning electron microscope (EVO LS15; Carl Zeiss, Jena, Germany).

## Authors' contributions

QHZ generated constructs over-expressing miR172a and miR172b, analyzed transformed plants and performed all molecular analyses. NMU provided pNU352 vector. QHZ, FG and CAH designed the experiments. QHZ and CAH wrote the manuscript. All authors read and approved the final manuscript.
